# The British Hospitals Association: A New Departure during War-Time

**Published:** 1916-10-07

**Authors:** T. Hayes

**Affiliations:** St. Bartholomew's Hospital


					"The British Hospitals Association: A New
Departure during War-Time."
To the. Editor of The Hospital.
Sir,?In the Editorial Note under the above heading
which appeared in your issue of September 2 it is sug-
gested that the statement made in the Report of the
Executive Committee of the British Hospitals Association
(August 1916) that the governing bodies of the hospital
nurse-training schools have no representation upon the
Council of the College of Nursing is inaccurate. If this
is so, I should be much obliged if you would kindly refer
me to the clause in the articles of association of the
College which provides for such representation.?I am,
Sir, yours faithfully,
T. Hayes.
St. Bartholomew's Hospital,
September 27, 1916.
[If Mr. Hayes will re-read the Editorial Note to -which
he objects (The Hospital, September 2, p. 516), he will
see that no suggestion was made therein that the govern-
ing bodies of the hospital nurse-training schools are
represented on the Council of the College of Nursing.
What was stated was that a proportion of the .Council of
the College are to be representatives of the nurse-
training schools, and this statement is absolutely accu-
rate.?Ed. The Hospital.]

				

